# Activity‐dependent redistribution of Kv2.1 ion channels on rat spinal motoneurons

**DOI:** 10.14814/phy2.13039

**Published:** 2016-11-24

**Authors:** Shannon H. Romer, Adam S. Deardorff, Robert E. W. Fyffe

**Affiliations:** ^1^ Department of Neuroscience, Cell Biology and PhysiologyBoonshoft School of MedicineWright State UniversityDaytonOhio

**Keywords:** C‐boutons, Kv2.1, voltage‐gated ion channels, activity dependent, *α*‐motoneuron

## Abstract

Homeostatic plasticity occurs through diverse cellular and synaptic mechanisms, and extensive investigations over the preceding decade have established Kv2.1 ion channels as key homeostatic regulatory elements in several central neuronal systems. As in these cellular systems, Kv2.1 channels in spinal motoneurons (MNs) localize within large somatic membrane clusters. However, their role in regulating motoneuron activity is not fully established in vivo. We have previously demonstrated marked Kv2.1 channel redistribution in MNs following in vitro glutamate application and in vivo peripheral nerve injury (Romer et al., 2014, *Brain Research*, 1547:1–15). Here, we extend these findings through the novel use of a fully intact, in vivo rat preparation to show that Kv2.1 ion channels in lumbar MNs rapidly and reversibly redistribute throughout the somatic membrane following 10 min of electrophysiological sensory and/or motor nerve stimulation. These data establish that Kv2.1 channels are remarkably responsive in vivo to electrically evoked and synaptically driven action potentials in MNs, and strongly implicate motoneuron Kv2.1 channels in the rapid homeostatic response to altered neuronal activity.

## Introduction

The intrinsic membrane properties of neurons in the central nervous system are controlled, in part, by the tight regulation of membrane‐bound ion channels. The localization of ion channels within certain membrane compartments and/or signaling ensembles is critical to synaptic integration and shaping of firing properties (Deardorff et al. 2013, 2014; Romer et al. 2014). In spinal motoneurons (MNs), as in other cell types, intrinsic membrane properties can be dynamically modified by changes in neuronal activity and pathology (Kuno et al. 1974a,b; Gustafsson and Pinter 1984; Foehring et al. 1986a,b; Wolpaw and Tennissen 2001; Bichler et al. 2007a; Meehan et al. 2010; Prather et al. 2011; Quinlan et al. 2011; Johnson et al. 2013). Identifying the responsible conductances and related ion channel expression patterns is critical to understanding MN physiology and pathology.

In mammalian MNs, Kv2.1 channels, which underlie delayed rectifier potassium currents, form distinct clusters that assemble at a variety of cellular locations, including highly regulated signaling ensembles at C‐bouton synaptic sites (Deng and Fyffe 2004; Muennich and Fyffe 2004; Wilson et al. 2004; Deardorff et al. 2013, 2014; Mandikian et al. 2014; Romer et al. 2014). In several neuronal systems, these unique ion channels undergo essential activity‐dependent changes in anatomic and physiologic parameters (Cudmore and Turrigiano 2004; Misonou et al. 2004, 2008; Surmeier and Foehring 2004; Mohapatra et al. 2009; Kihira et al. 2010; Misonou 2010; Nataraj et al. 2010; Deardorff et al. 2014; Romer et al. 2014). For example, in the highly clustered configuration observed in hippocampal and cortical pyramidal cells, Kv2.1 channels are phosphorylated and have high activation and deactivation thresholds together with slow kinetics (Murakoshi et al. 1997; Misonou et al. 2004, 2005; Surmeier and Foehring 2004; Mohapatra and Trimmer 2006; Misonou 2010; Guan et al. 2013; Liu and Bean 2014). With prolonged excitatory drive, Ca^2+^/calcineurin‐dependent pathways accelerate Kv2.1 channel kinetics and lower Kv2.1 channel activation/deactivation thresholds to homeostatically reduce neuronal firing rate (Surmeier and Foehring 2004; Park et al. 2006; Mohapatra et al. 2009). At the same time, Kv2.1 channels rapidly decluster in the membrane, providing a biomarker for channel physiological that can be measured in immunohistological sections (Surmeier and Foehring 2004; Park et al. 2006; Mohapatra et al. 2009; Romer et al. 2014).

Recently, we proposed that the dynamic reorganization of delayed rectifier Kv2.1 channels in mammalian MNs plays a critical role in adjusting input‐output gain in response to prolonged physiologic or pathologic excitatory drive (Deardorff et al. 2014). In support, we have shown that MN Kv2.1 channels dramatically and significantly decluster following glutamate application in vitro and peripheral nerve injury in vivo (Romer et al. 2014). These data strongly indicate MN Kv2.1 channels have the capacity to rapidly and dynamically respond to altered MN activity.

Here, we extend these findings using direct electrical stimulation of peripheral nerves in vivo to demonstrate that Kv2.1 clustering in MNs is activity dependent in the uninjured, adult animal. Moreover, we demonstrate that sensory‐evoked synaptic inputs to MNs also contribute to Kv2.1 clustering dynamics. These observations are critical for interpreting activity‐dependent intrinsic modifications in a variety of physiological and pathological states.

## Experimental Procedures

### Animal use

All animal procedures were performed according to National Institutes of Health (NIH) guidelines and reviewed by the local Laboratory Animal Use Committee at Wright State University. Detailed immunohistochemical analysis of Kv2.1 channel expression was performed on adult female (230–250 g) Sprague–Dawley rats (*n* = 24) following retrograde labeling of medial gastrocnemius (MG) MNs and subsequent in vivo sciatic nerve stimulations or sham control experiments. All survival and terminal surgeries were performed with rats deeply anesthetized (absent withdrawal and corneal reflex) by isoflurane inhalation (induction 4–5%; maintenance 1–3%, both in 100% O_2_).

### Retrograde tracer

All rats in this study underwent a single sterile survival surgery to retrogradely label MG MNs for post hoc identification (Romer et al. 2014). The triceps surae were exposed by a midline incision through the skin and biceps femoris muscle of the left hindlimb. A total of 25 *μ*L of 0.5% Cholera Toxin Subunit B‐555 (CTB, Invitrogen, Carlsbad, CA) was administered throughout the MG muscle by a series of small injections. The wound was irrigated and closed in layers. Animals received 0.1 mL of 0.3 mg/mL buprenorphine every 12 h for postoperative pain medication for 48 h and were monitored closely by professional staff.

### In vivo sciatic stimulation

After adequate time for retrograde transport of CTB (>7 days), the effects of nerve activity on MN Kv2.1 channel clustering were examined. Standard procedures were used to prepare the left hindlimb and, when necessary, the lumbar spinal roots for electrophysiological stimulation or rhizotomy (Seburn and Cope 1998; Haftel et al. 2004, 2005; Bullinger et al. 2011a). Rats were deeply anesthetized and vital signs were closely monitored. Respiratory rate (40–60), end‐tidal CO_2_ (3–5%), oxygen saturation (>90%), heart rate (300–500 beats/min), and core temperature (36–38°C) were maintained by adjusting isoflurane concentration and/or radiant heat. A small midline incision was made through the skin and biceps femoris of the left hindlimb to expose the left sciatic nerve, which contains axons destined for the MG nerve in the popliteal fossa. With careful surgical dissection, the sciatic nerve was freed from surrounding tissue and placed in a bipolar cuff electrode. Experiments proceeded with one of the terminal studies described below. Optimal parameters for nerve stimulation were set with the following considerations: (a) stimulus frequency (100 Hz) approximates the upper limit of the MN firing rate in anesthetized rats (Hamm et al., 2010; Turkin et al., 2010; Obeidat et al. 2014); and (b) stimulus strength (4X MG contraction threshold) maximally activates large diameter axons, including motor axons and Group I/II afferents (Bichler et al. 2007b; Bullinger et al. 2011b).

### Sciatic nerve stimulation

To study the effects of nerve activity on MN Kv2.1 channel clustering, the left sciatic nerve in three animals (*n* = 3) was, stimulated for 10 min, and <5 min later the animals were sacrificed for histological analysis. For all analyses, Kv2.1 channel clustering in stimulated MG MNs were compared to MG MNs in three sham control animals (*n* = 3), in which the sciatic nerve was exposed but not stimulated.

To verify the effects of nerve stimulation on MN Kv2.1 channel clustering are dependent on the centripetal conduction of action potentials, 0.5 *μ*mol/L Tetrodotoxin (TTX), a voltage‐gated sodium (Na_V_) channel blocker, was applied to the sciatic nerve in three animals (*n* = 3) proximal to the stimulation site to block action potential generation and propagation. The sciatic nerve was subsequently stimulated for 10 min, and <5 min later the animals were sacrificed for histological analysis. To assess any confounding effects of TTX treatment on Kv2.1 channel clustering, the sciatic nerve of an additional animal was exposed and treated with 0.5 *μ*mol/L TTX for 10 min without nerve stimulation.

To determine if changes in Kv2.1 channel clustering that occur following nerve stimulation are reversible, the left sciatic nerve in three animals (*n* = 3) was stimulated for 10 min (as in experiment 1). Animals remained under isoflurane anesthesia and 2 h later were sacrificed for histological analysis.

### Selective dorsal rhizotomy

To separate the effects of motor nerve stimulation from sensory‐evoked central synaptic activity on MN Kv2.1 channel clustering, a selective dorsal rhizotomy (SDR) was performed in three rats (*n* = 3) prior to sciatic nerve stimulation. Following surgical dissection of the left hindlimb and lower back, rats were fixed prone in a rigid recording frame. Skin flaps were used to construct pools for bathing all exposed tissues with warm mineral oil. Dorsal exposure of the lumbosacral spinal cord by laminectomy and longitudinal incision of the dura mater provided access to lumbosacral dorsal roots (L1–S2), which were carefully dissected free of surrounding tissue and suspended in continuity on bipolar silver hook electrodes for recording. Dorsal roots containing sciatic nerve afferents were electrophysiologically identified and cut near their entry to the spinal cord on the left side. Remaining dorsal roots were similarly cut. The left sciatic nerve was subsequently stimulated for 10 min, and <5 min later the animal was sacrificed for histological analysis.

Two methods were employed to control for confounding effects of SDR on Kv2.1 channel clustering. First, a pair of fine‐wire silver electrodes was inserted into the MG muscle for electromyography (EMG) recording. EMG records demonstrated no detectable MG electrical activity during SDR, indicating any spontaneous discharge of cut afferents did not bring a sufficient number of MG MNs to threshold. Second, three additional animals (*n* = 3) underwent the same dorsal rhizotomy described above. These animals were then sacrificed for histological analysis without sciatic nerve stimulation.

### Selective dorsal root stimulation

To isolate the effects of MN synaptic activation on MN Kv2.1 channel clustering, dorsal roots in three rats (*n* = 3) were directly stimulated. As described above, rats were fixed in a rigid recording frame and dorsal roots containing sciatic nerve afferents were identified and suspended in continuity on bipolar silver hook electrodes. Dorsal roots were subsequently stimulated at 100 Hz for 10 min (4X threshold for a visible dorsal root volley following sciatic nerve stimulation), and <5 min later the animal was sacrificed for histological analysis.

### Immunohistochemistry

Immediately following stimulations and under anesthesia, rats received intraperitoneal overdose of pentobarbital (150 mg/kg i.p.). All rats were transcardially perfused with a vascular rinse followed with 4% paraformaldehyde in 0.1 mol/L phosphate buffer at pH 7.3. The spinal cords were removed, postfixed for 2 h in 4% paraformaldehyde and cryoprotected overnight in 15% sucrose in 0.1 mol/L phosphate buffer (≈300 mOsm). Transverse sections (50 *μ*m) were obtained from the L4 and L5 spinal cord segments on a cryostat and immunostained free floating. Kv2.1 immunocytochemistry was performed, using mouse anti‐Kv2.1 clone D4/11 (catalog number 75‐047) at 1:1000 in PBS with 0.1% Triton X pH 7.3 that was developed and/or obtained from the UC Davis/NINDS/NIMH Neuromab facility, supported by NIH grant U24NS0506060 and maintained by the Department of Pharmacology, School of Medicine, University of California, Davis, CA. Immunoreactivity was detected with species‐specific secondary antibodies conjugated to Alexa 488 (Jackson Immuno, West Grove, PA). Nissl immunocytochemistry was performed, using blue fluorescent nissl (1:100, Molecular Probes, Carlsbad, CA).

### Confocal microscopy and quantitative analysis

As previously described (Romer et al. 2014), micrographs of immunolabeled lumbar *α*MN images were obtained on an Olympus Fluoview 1000 (Center Valley, PA) confocal microscope with a 60x oil immersion objective (N.A 1.35) at 1.0 *μ*m *z*‐steps. For every ipsilateral CTB‐labeled MG *α*MN imaged and analyzed, a contralateral *α*MN was also selected of the same approximate size and position within the motor pools as an internal bilateral control.

Image stacks were quantitatively analyzed for Kv2.1‐IR cluster areas as previously described (Muennich and Fyffe 2004) in Image Pro Software (Media Cybernetics, Silver Springs, MD). Briefly, en face Kv2.1 immunoreactive (IR) macroclusters (diameter >1.0 *μ*m) were measured in single optical sections on the soma of MNs that innervate the MG, as revealed by retrograde labels (Romer et al. 2014).

### Figure composition

Microscope images were prepared by adjusting contrast and brightness in Image Pro Plus Software (Media Cybernetics, Bethesda, MD) and always preserved all the information content of the images. Figures were composed, using CorelDraw (v. 12.0). Graphs were composed in SigmaPlot (version 9.0, Systat Software, SPSS Inc, Chicago, IL). Some images were sharpened using a “high gauss” filter in image pro. Quantification was always carried out in original unprocessed images.

### Statistics

All statistical tests were performed in SigmaPlot (version 9.0, Systat Software, SPSS Inc) software. Nonparametric Mann–Whitney Wilcoxon rank sum tests were used in all pairwise comparisons. Statistical significance was set at *P = *0.05 and data presented in figures is mean ± SD.

## Results

### Redistribution of lumbar motoneuron Kv2.1 is activity dependent

**Figure 1:**
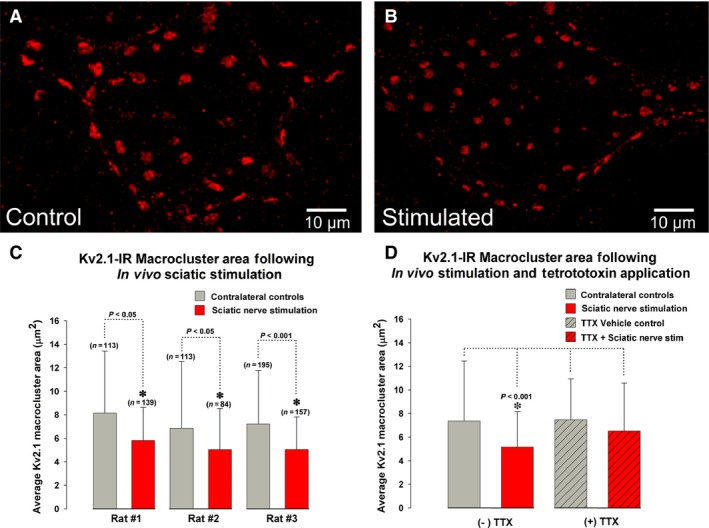
Reorganization of Kv2.1‐IR clusters on rat lumbar *α*‐motoneurons is activity dependent. The sciatic nerve was stimulated in vivo at 150 Hz and 4x tetanic threshold for 10 min. Motoneurons displayed in (A and B) are from the same tissue slice with fixed imaging parameters. Scale bars are 10 *μ*m. (A) Micrograph of a confocal stack (33 × 1.0 *μ*m *z*‐steps) of a contralateral “control” motoneuron showing Kv2.1‐IR (red). (B) Micrograph of a confocal stack (30 × 1.0 *μ*m *z*‐steps) of an MG *α*‐motoneuron following 10 min of sciatic nerve stimulation showing reduced Kv2.1‐IR (red) macrocluster areas compared to the unstimulated motoneuron in panel A. (C) Quantitative analysis of reduced Kv2.1‐IR soma macrocluster areas on medial gastrocnemius *α*‐motoneurons following 10 min of sciatic nerve stimulation in all 3 rats sampled. (D) Pooled quantitative analysis of Kv2.1‐IR following 0.5 *μ*mol/L tetrodotoxin (TTX) showing no significant changes compared to the absence of TTX application. (C and D) *N *= the number of Kv2.1 macroclusters sampled. Significance (*P* < 0.05) is indicated with asterisk and determined with Mann–Whitney and data are presented ± SD.

Previous work in a rat in vitro lumbar spinal cord slice preparation indicates lateral translocation, or declustering, of membrane Kv2.1 protein in MNs to more uniform distribution following glutamate treatment (Romer et al. 2014). These results suggest that in MNs, Kv2.1 clustering dynamics may be activity dependent. The goal of the experiments reported in this section was to evaluate the effects of nerve activity on Kv2.1‐IR clusters on MG MN somas, using an in vivo preparation in adult anesthetized rats to isolate the sciatic nerve for electrophysiological stimulation. Following 10 min of unilateral sciatic nerve stimulation, MN Kv2.1‐IR macrocluster area was 30% reduced (Fig. 1; control = 7.37 *μ*m^2^ ± 5.1 SD, *n* = 421 clusters in 3 rats versus stimulated = 5.15 *μ*m^2^ ± 3.01 SD, *n* = 380 clusters in 3 rats, *P* < 0.001, Mann–Whitney test). On a cell by cell basis, when the average Kv2.1 macrocluster area per MN was examined, there was a significant 35% reduction (control = 7.95 *μ*m^2^ ± 2.4 SD, *n* = 42 MNs in 3 rats versus stimulated = 5.13 *μ*m^2^ ± 1.3 SD, *n* = 38 MNs in 3 rats, *P* < 0.001, Mann–Whitney test). No significant differences were found in sham control rats compared to the contralateral, unstimulated, side of the experimental rats. The reduction in macrocluster area observed here is consistent with similar observations made in MNs and other neuronal types (Misonou et al. 2004, 2005; Mohapatra et al. 2009; Romer et al. 2014).

To confirm that Kv2.1 channel dynamic clustering is a direct result of the sciatic nerve stimulation and not a confounding effect from surrounding tissue damage, 0.5 *μ*mol/L tetrodotoxin (TTX) was applied directly to the sciatic nerve proximal to the stimulation site to block centripetal action potential propagation. The application of both TTX alone and TTX with subsequent sciatic nerve stimulation did not affect the Kv2.1‐IR macrocluster sizes on MG MN somas (Fig. 1D; TTX Control = 7.45 *μ*m^2^ ± 3.5 SD, *n* = 157 clusters vs. TTX + Stimulation = 7.75 *μ*m^2^ ± 3.34 SD, *n* = 637 clusters). These results indicate that MN Kv2.1‐IR declustering is a result of the electrophysiological stimulation of the sciatic nerve, and validate that Kv2.1 clustering dynamics are activity dependent in rat lumbar MNs.

### Activity‐dependent changes in Kv2.1 are reversible

**Figure 2:**
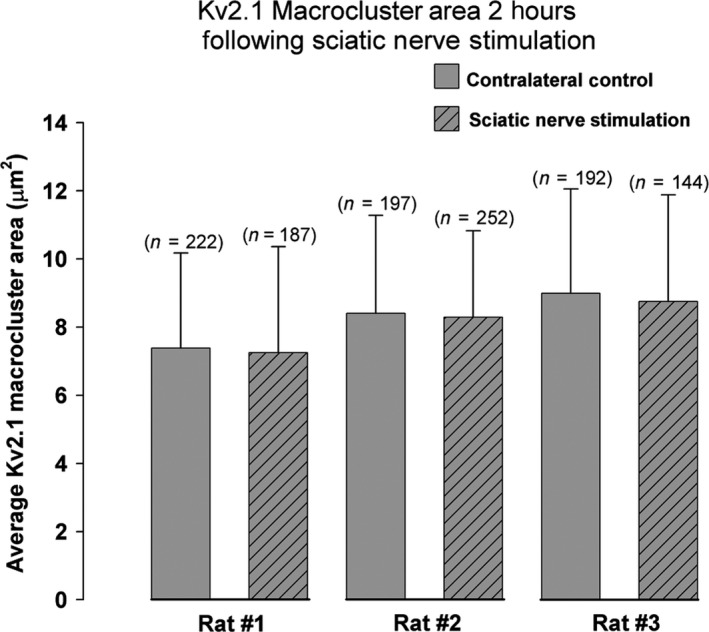
Kv2.1‐IR macroclusters on medial gastrocnemius *α*‐motoneurons recover to control sizes by 2 h following sciatic nerve stimulation in all 3 rats sampled. Absence of significance was determined with Mann–Whitney *T*‐test and data are presented ± SD. *N *= number of Kv2.1 macroclusters sampled.

Following peripheral nerve injury, significant and persistent differences were observed in MN Kv2.1‐IR macrocluster area over weeks to months (Romer et al. 2014). To determine if the activity‐dependent impact on MN Kv2.1‐IR channel clustering persists or is reversed upon stimulus removal, the sciatic nerve was isolated and stimulated under the same parameters previously described (see Methods: In Vivo *Sciatic Stimulation*). Following 10 min of nerve stimulation, these rats remained anesthetized for an additional 2 h prior to sacrifice and subsequent harvesting of the lumbar spinal cord. No significant differences were observed in Kv2.1‐IR macrocluster sizes in MG MNs 2 h after stimulation compared to internal bilateral controls (Fig. 2; control = 8.22 *μ*m^2^ ± 3.0 SD, *n* = 611 clusters, 47 MNs vs. stimulated = 8.07 *μ*m^2^ ± 2.9 SD, *n* = 583 clusters, 50 MNs in 3 rats). We also observed that Kv2.1‐IR macrocluster size on the contralateral control side appeared to be larger following 2 h of anesthesia compared to those rats that were anesthetized for less than 20 min suggesting that prolonged inactivity under anesthesia may impact Kv2.1‐IR clusters. With less than 20 min deep isoflurane anesthesia, Kv2.1 macroclusters were on average 7.765 *μ*m^2^ ± 4.1 SD (*n* = 979 clusters in 6 rats). However, with 2 h of deep isoflurane anesthesia, Kv2.1 macroclusters were on average 8.10 *μ*m^2^ ± 3.4 SD (*n* = 1157 clusters, 5 rats; *P* = 0.04 Mann–Whitney). Altogether, these results demonstrate Kv2.1 declustering is quickly reversible (within 2 h) upon stimulus removal.

### Motor axon stimulation and sensory‐evoked synaptic activation impacts Kv2.1‐IR channel clustering on motoneuron somas

**Figure 3:**
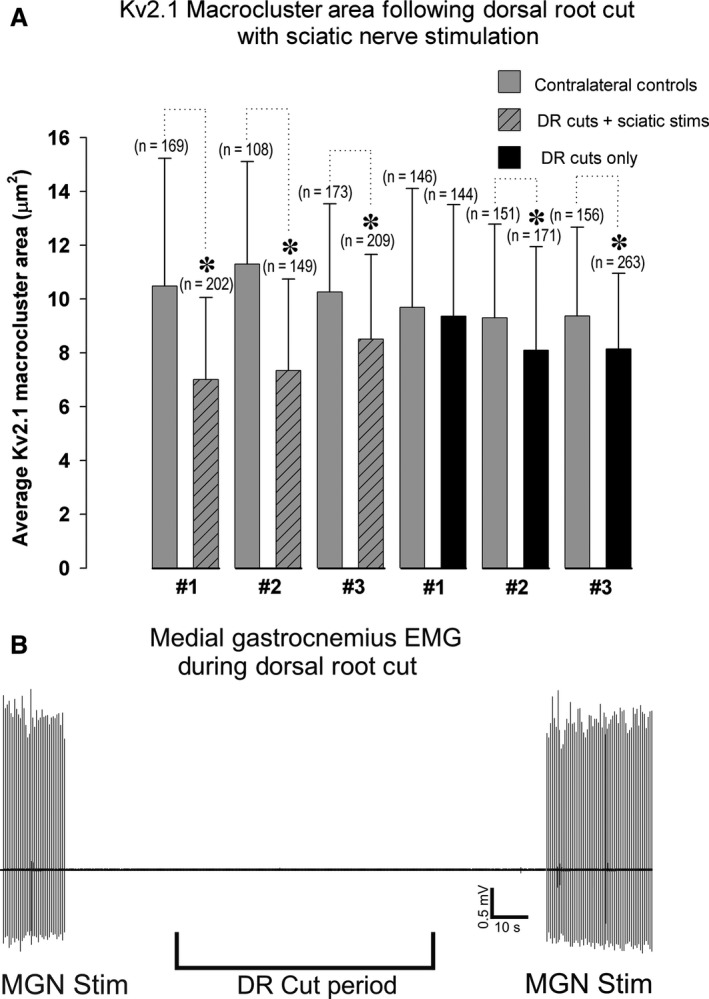
Kv2.1‐IR macroclusters on *α*‐motoneurons significantly reduce when motor axons are stimulated. (A) Quantitative analysis of reduced Kv2.1‐IR soma macrocluster areas on lumbar *α*‐motoneurons following sciatic nerve stimulation with dorsal rhizotomy in all 3 rats sampled. The impact of dorsal rhizotomy on Kv2.1‐IR macrocluster areas on lumbar *α*‐motoneurons was quantified in 3 rats. *N *= the number of Kv2.1 macroclusters sampled. Significance (*P* < 0.05) is indicated with asterisk and determined with Mann–Whitney *T*‐test and data are presented ± SD. (B) Electromyography (EMG) of medial gastrocnemius (MG) muscle indicates that the injury discharge during dorsal root cuts did not cause motor output activity in the muscle. The MG nerve (MGN) itself was stimulated before and after the dorsal roots were cut to confirm EMG recordings.

During sciatic nerve stimulation, not only are MN axons antidromically driven, but the stimulus also activates sensory circuits that can evoke synaptic potentials onto MNs. An SDR was therefore performed to isolate effects of motor axon stimulation on MN Kv2.1‐IR clustering dynamics from those of sensory‐evoked synaptic activity. With the motor axon volley isolated and the sciatic nerve stimulated with the same fixed parameters described above (see Experimental Procedures: In Vivo *Sciatic Stimulation*) there was a significant ≈28% reduction in Kv2.1 macrocluster areas on MG MNs compared to internal bilateral controls (Fig. 3; control = 10.60 *μ*m^2^ ± 4.0 SD, *n* = 450 clusters in 41 MNs vs. antidromic stimulation = 7.66 *μ*m^2^ ± 3.25 SD, *n* = 560 clusters in 44 MNs in 3 rats, *P* < 0.001, Mann–Whitney test).

To control for the injury discharge from the dorsal rhizotomy (Eschenfelder et al. 2000; Sun et al. 2005), the Kv2.1 cluster sizes were analyzed in rhizotomized rats without nerve stimulations and EMG records were taken from the MG muscle during the rhizotomy procedure. Following dorsal rhizotomy, we observed an approximately 11% significant reduction in Kv2.1‐IR macrocluster area (Fig. 3; control = 9.45 *μ*m^2^ ± 3.2 SD, *n* = 453 clusters in 40 MNs vs. dorsal rhizotomy = 8.43 *μ*m^2^ ± 3.54 SD, *n* = 578 clusters in 40 MNs in 3 rats, *P* < 0.001, Mann–Whitney test). These results suggest that the dorsal rhizotomy itself has an impact on MN Kv2.1 clustering. However, EMG records of all 6 rats analyzed show the injury discharge of dorsal root afferents was not sufficient to bring MNs to threshold (Fig. 3B).

**Figure 4:**
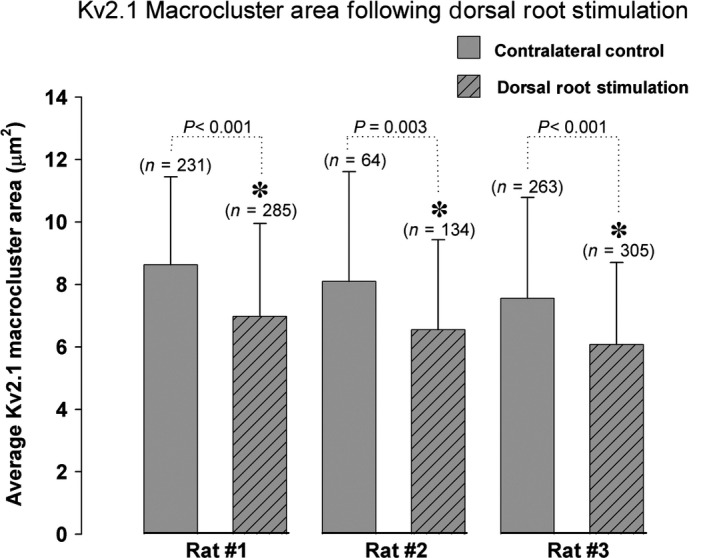
Kv2.1‐IR macroclusters on *α*‐motoneurons significantly reduce when sensory afferent circuitry is driven through dorsal root stimulations. Quantitative analysis of Kv2.1‐IR on medial gastrocnemius *α*‐motoneuron somas in all three animals samples demonstrates significant decrease in area. *N *= the number of Kv2.1 macroclusters sampled. Significance (*P* < 0.05) is indicated with asterisk and determined with Mann–Whitney *T*‐test and data are presented ± SD.

Alternatively, to determine if sensory‐evoked synaptic input could impact Kv2.1‐IR channel clustering on MG MNs, all dorsal roots containing sciatic nerve afferents were isolated and stimulated at 100 Hz to selectively drive sensory circuits. In all rats tested, 10 min of dorsal root stimulation caused a significant 19% reduction in Kv2.1‐IR macrocluster areas on MN somas (Fig. 4, control = 7.46 *μ*m^2^ ± 3.1 SD, *n* = 558 clusters in 36 MNs vs. dorsal root stimulation = 5.97 *μ*m^2^ ± 2.8 SD, *n* = 724 clusters in 39 MNs in 3 rats, *P* < 0.001, Mann–Whitney test). Altogether, results in this section demonstrate that both motor nerve activity and sensory‐evoked synaptic activity can induce Kv2.1‐IR channel declustering in MNs.

## Discussion

**Figure 5:**
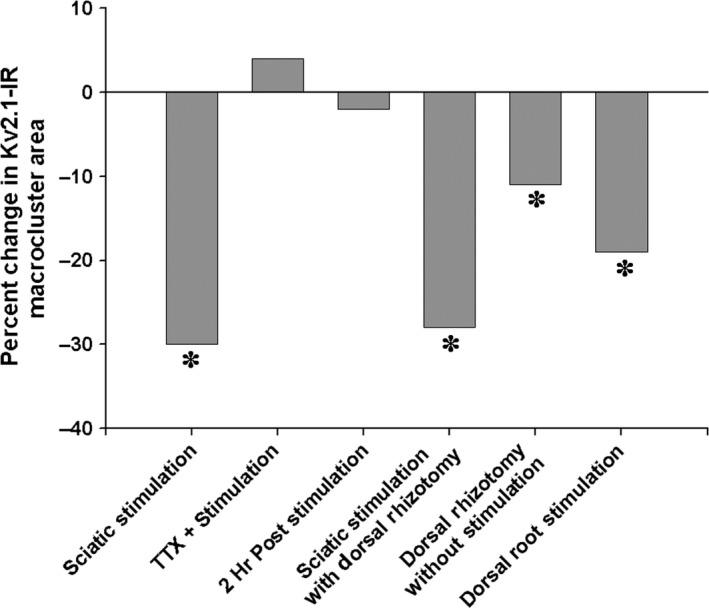
Summary of spinal motoneuron Kv2.1 ion cluster responses to various in vivo stimuli. Kv2.1 ion channels significantly reduce by 30% following sciatic nerve stimulation (sciatic stimulation), an effect inhibited by the application of tetrodotoxin proximal to the stimulation site (TTX + stimulation) that results in an 4% increase in cluster size. Channel cluster sizes are restored to the original sizes, with a 2% reduction, 2 h following the sciatic nerve stimulation (2 h poststimulation). A significant 28% reduction in cluster size was produced when the effect of the sciatic nerve stimulation was isolated to just antidromic activation of motor axons through dorsal rhizotomy (sciatic stimulation with dorsal rhizotomy). However, the dorsal rhizotomy itself, without nerve stimulation, also induced a significant 11% reduction cluster areas (dorsal rhizotomy without stimulation). Finally, the sensory‐evoked synaptic activity, through dorsal root stimulations (dorsal root stimulation), caused a significant 19% reduction in cluster areas.

The striking membrane organization of Kv2.1 channels in rodent lumbar MNs together with their strategic positioning at specific synapses within signaling ensembles (Muennich and Fyffe 2004; Deardorff et al. 2013, 2014), make them a valuable model for the study of channel regulation under conditions of altered activity. We have previously shown that both in vitro application of glutamate and in vivo axotomy induce rapid Kv2.1 cluster dispersal in lumbar spinal MNs, suggesting clustering dynamics may be activity‐dependent (Romer et al. 2014). Here, we use an in vivo preparation that provides direct control of electrophysiological activity in intact, adult rodents to evaluate activity‐dependent changes in MN Kv2.1 channels. Our results demonstrate that MN Kv2.1 ion channels are dynamically responsive to the diverse set of stimuli employed in these experimental paradigms (Fig. 5). While it is interesting to consider the relative contribution of motor versus sensory nerve activity on Kv2.1 cluster sizes in these experiments, caution must be taken in interpreting relative effects from these data. Nevertheless, several trends are, of course, evident. First, the cumulative effect of motor and sensory nerve stimulation produced the most dramatic decrease in Kv2.1 cluster size. Although this mode of motoneuron activation is not physiological, this experimental approach is the only one to achieve controlled, in vivo activation of the MNs under investigation and may in fact underestimate the effect of antidromic stimulation on Kv2.1 channel declustering, due to coactivation of Renshaw cell recurrent inhibitory circuits (Obeidat et al. 2014). Second, the antidromic stimulation of sciatic motor axons, following selective rhizotomy of sciatic dorsal roots, is more effective at declustering Kv2.1 channels than stimulating sciatic dorsal roots alone. However, dorsal root rhizotomy itself causes declustering of Kv2.1 in MNs, indicating that the extent of Kv2.1 channel declustering observed following sciatic nerve stimulation with dorsal root rhizotomy cannot be solely attributed to the antidromic activity of sciatic motor axons. To achieve more physiologically relevant, synaptically driven activation of MNs, we selectively stimulated sciatic dorsal roots with a stimulus strength designed to maximally activate large diameter axons, including Group I/II proprioceptive afferents (Bichler et al. 2007b; Bullinger et al. 2011b). The observed declustering of Kv2.1 channels, we presume, is primarily driven by direct 1a afferent – motoneuron synaptic activity. However, the influence of other, polysynaptic excitatory and inhibitory segmental spinal circuits is unknown and cannot be ignored. Despite this difficulty in quantifying differential effects of sensory versus motor nerve stimulation on Kv2.1 clustering dynamics, our data conclusively demonstrate that Kv2.1 channels rapidly respond to increases in neuronal activity. Below, we discuss the likely impact of Kv2.1 channel clustering on motoneuron firing behavior and the manner in which this unique channel may be integrated within the C‐Bouton signaling ensemble.

### An intrinsic neuronal mechanism for homeostatic plasticity

Homeostatic mechanisms contribute to stabilizing the activity of neurons and neuronal circuits to maintain optimal firing levels (Katz and Shatz 1996; Burrone and Murthy 2003; Murphy 2003; Thiagarajan et al. 2005; Marder and Goaillard 2006; Walmsley et al. 2006; Turrigiano 2011, 2012; Ganguly and Poo 2013; O'Leary et al. 2014). Because neuronal firing arises from the interplay of synaptic currents and intrinsic membrane properties, homeostatic mechanisms may specifically target numerous ion channels or synaptic proteins to optimize and adapt firing rate to meet different demands (Marder and Goaillard 2006; Turrigiano 2011). Multiple intrinsic homeostatic mechanisms, activated by intracellular calcium, have been shown to directly influence ion channels and can lead to rapid dramatic changes in neuronal firing patterns (Marder and Goaillard 2006; O'Leary et al. 2014; Turrigiano et al., 1994). In other neuronal types, it has been demonstrated that Kv2.1 ion channels are likely heavily involved in homeostatic plasticity (Surmeier and Foehring 2004; Park et al. 2006; Mohapatra et al. 2009). Specifically, following prolonged excitatory drive, Ca^2+^/calcineurin‐dependent pathways accelerate Kv2.1 channel kinetics and lower Kv2.1 channel activation and deactivation thresholds to homeostatically reduce neuronal firing rate. The rapid (10 min) activity‐dependent alterations in MN Kv2.1 channel clustering dynamics shown here is consistent with these observations and those made in hippocampal and cortical pyramidal neurons that calcium‐dependent dephosphorylation pathways posttranslationally regulate Kv2.1 clustering and gating kinetics (Park et al. 2006; Mohapatra et al. 2009). Given that Kv2.1 channel clustering provides an anatomical window into channel physiology (Misonou et al. 2004, 2008; Mohapatra et al. 2009; Misonou 2010), this study supports the hypothesis that Kv2.1 channels provide a calcium‐dependent, intrinsic neuronal mechanism for homeostatic reductions in the firing rate (Cudmore and Turrigiano 2004; Misonou et al. 2004, 2008; Surmeier and Foehring 2004; Mohapatra et al. 2009; Kihira et al. 2010; Misonou 2010; Nataraj et al. 2010; Deardorff et al. 2014; Romer et al. 2014). Moreover, the time course of the MN Kv2.1 channel response is likewise consistent with observations that intrinsic homeostatic mechanisms can respond more rapidly than synaptic mechanisms (Karmarkar and Buonomano 2006).

Many electrophysiological and modeling studies show that MN intrinsic properties respond profoundly to both pathologic and nonpathologic alterations in activity (Kuno et al. 1974a,b; Gustafsson and Pinter 1984; Foehring et al. 1986a,b; Wolpaw and Tennissen 2001; Bichler et al. 2007a; Meehan et al. 2010; Prather et al. 2011; Quinlan et al. 2011; Johnson et al. 2013). However, only a few motoneuron studies have characterized the ion channel alterations that could underlie these effects, and of these studies, most examined transcriptional expression changes over days to weeks (Anneser et al. 1999, 2000; Alvarez and Fyffe 2007; Woodrow et al. 2013). Importantly, the MN Kv2.1 channel clustering dynamics shown here are unlikely to be a product of transcriptional regulation but rather are consistent with a more rapid mechanism of cellular plasticity that is “built‐in” to the phosphorylation state of the channel itself, as is likely the case for Cav1.3 channels underlying persistent inward calcium currents (Brownstone 2006; Heckman et al. 2008).

Kv2.1 cluster sizes rapidly return to their original sizes within 2 h following the cessation of nerve stimulation. Whether these clusters are physically “reclustering” or are the result of protein turn‐over restoring original cluster sizes is unknown. However, that the cluster sizes rapidly restore upon stimulus removal is further support that Kv2.1 channels are homeostatically driven to respond to alterations in activity. These findings are especially important when interpreting long‐term pathological changes in Kv2.1 channel clustering. For instance, following peripheral nerve injury, MN Kv2.1‐IR macrocluster areas were reduced over weeks to months (Romer et al. 2014), suggesting the continued existence of excitatory drive.

### The role of Kv2.1 at the C‐bouton

The largest Kv2.1 clusters are localized within a signaling ensemble at C‐bouton synapses (Deardorff et al. 2014), important cholinergic loci for state‐dependent modifications of MN firing rate (Miles et al. 2007; Zagoraiou et al. 2009). Our hypothesis on the modulatory effects of cholinergic signaling in *α*MNs is centered on a highly regulated system surrounding a Ca^2+^ microdomain for the precise and nuanced regulation of cell firing (Deardorff et al. 2014). Specifically, activation of the muscarinic m2 receptors, concentrated at C‐boutons, likely inhibit Ca^2+^ currents to prevent activation of Ca^2+^/calcineurin‐dependent pathways and thus maintain Kv2.1 clustering. However, if prolonged/pathologic excitatory drive causes large changes in intracellular Ca^2+^ sufficient to allow diffusion of Ca^2+^ from neighboring compartments, there would be rapid Kv2.1 channel declustering, to homeostatically suppress repetitive firing (Deardorff et al. 2014; Romer et al. 2014). While the impact of Kv2.1 channel clustering on MN physiology is still undefined, our observations of Kv2.1 synaptic reorganization at C‐boutons following prolonged excitatory drive offer support to their potential role at the C‐bouton.

## Conclusion

The central nervous system is remarkable in its capacity to respond and adapt to environmental demands through diverse activity‐dependent mechanisms. One mechanism that is critical for the homeostatic regulation of several neuronal systems is the highly clustered, somato‐dendritic Kv2.1 channel. Here, we show for the first time, using a fully intact and in vivo rat preparation, that Kv2.1 channel clustering dynamics in lumbar MNs (a) are activity‐dependent, (b) are responsive to both intrinsic and synaptically driven changes in MN activity, and (c) reverse (within 2 h) upon stimulus removal. Altogether, these results further support the hypothesis that the activity‐dependent properties of Kv2.1 ion channels provide an intrinsic mechanism for the homeostatic regulation of MN firing properties (see also Deardorff et al. 2014; Romer et al. 2014).

## Conflicts of Interest

The authors declare no conflicts of interest, financial or otherwise.
